# Clinical practice guidelines for the diagnosis and management of Duchenne muscular dystrophy: a scoping review

**DOI:** 10.3389/fneur.2023.1260610

**Published:** 2024-01-05

**Authors:** Marco Malaga, Aaron Rodriguez-Calienes, Fabian A. Chavez-Ecos, Andrely Huerta-Rosario, Giancarlo Alvarado-Gamarra, Miguel Cabanillas-Lazo, Paula Moran-Ballon, Victor Velásquez-Rimachi, Peggy Martinez-Esteban, Carlos Alva-Diaz

**Affiliations:** ^1^Facultad de Medicina Humana de la Universidad de San Martín de Porres, Lima, Peru; ^2^Red de Eficacia Clínica y Sanitaria, REDECS, Lima, Peru; ^3^Grupo de Investigación Neurociencia, Efectividad Clínica y Salud Pública, Universidad Científica del Sur, Lima, Peru; ^4^Sociedad Científica de Estudiantes de Medicina de Ica, Universidad Nacional San Luis Gonzaga, Ica, Peru; ^5^Facultad de Medicina Humana, Universidad Nacional Federico Villarreal, Lima, Peru; ^6^Instituto de Investigación Nutricional, Lima, Peru; ^7^Hospital Nacional Edgardo Rebagliati Martins, Lima, Peru; ^8^Sociedad Científica de San Fernando, Lima, Peru; ^9^Sociedad Científica Universidad San Martín de Porres, Lima, Peru; ^10^Instituto Nacional de Salud del Niño, San Borja, Lima, Peru; ^11^Universidad Señor de Sipán, Chiclayo, Peru; ^12^Servicio de Neurología, Departamento de Medicina y Oficina de Apoyo a la Docencia e Investigación (OADI), Hospital Daniel Alcides Carrión, Callao, Peru

**Keywords:** Duchenne muscular dystrophy, rare diseases, practice guidelines, evidence-based practice, review

## Abstract

**Introduction:**

Our objective was to identify recent CPGs for the diagnosis and management of DMD and summarize their characteristics and reliability.

**Methods:**

We conducted a scoping review of CPGs using MEDLINE, the Turning Research Into Practice (TRIP) database, Google Scholar, guidelines created by organizations, and other repositories to identify CPGs published in the last 5 years. Our protocol was drafted using the Preferred Reporting Items for Systematic Reviews and Meta-analyses for scoping reviews. To assess the reliability of the CPGs, we used all the domains included in the Appraisal of Guidelines Research and Evaluation II.

**Results:**

We selected three CPGs published or updated between 2015 and 2020. All the guidelines showed good or adequate methodological rigor but presented pitfalls in stakeholder involvement and applicability domains. Recommendations were coherent across CPGs on steroid treatment, except for minor differences in dosing regimens. However, the recommendations were different for new drugs.

**Discussion:**

There is a need for current and reliable CPGs that develop broad topics on the management of DMD and consider the challenges of developing recommendations for RDs.

## 1 Introduction

Duchenne muscular dystrophy (DMD) is a rare disease (RD) primarily affecting males due to its association with the X-linked chromosome (1, 2). DMD arises from a mutation in the DMD gene, leading to the absence or deficiency of the essential dystrophin protein (2). This genetic deficiency sets in motion a series of damaging processes within muscle cells, including oxidative stress injuries, imbalanced calcium regulation, and instability of the sarcolemma, these processes harm muscle cells, disrupt neuromuscular junction and abnormal differentiation of muscle satellite cells (2, 3). Collectively, these factors contribute to the progressive weakening and degeneration of muscles observed in individuals with DMD (2). The global prevalence and estimated birth prevalence of DMD are 7.1 and 19.8 male patients per 100,000 individuals, respectively (4). Unfortunately, many patients, especially in developing countries with inadequate standards of care, do not survive beyond their pediatric years (4).

For an RD such as DMD, the development of clinical practice guidelines (CPGs) involves multiple obstacles from evaluation and the synthesis of evidence to the formulation of recommendations and knowledge translation, and the data available may not be sufficient (5, 6). CPGs for the diagnosis and management of DMD could be useful as an aid to health care professionals for improving the quality of care and simultaneously reducing potentially harmful or ineffective interventions for patients (7). For RDs, CPGs are additionally useful for increasing transparency and allowing collaboration in improvements in patient care (8). For these reasons, guidelines for RDs must be prepared and provide recommendations with the best available evidence, even if it is considered insufficient.

Scoping reviews cover a broader field of review than systematic reviews (SRs); in addition, they can be used to qualitatively describe and evaluate the methodology of a CPG (9). Some similar papers were related to the diagnosis and treatment of liver tumors, diabetic macular edema and atopic dermatitis (10–12). Due to the importance of high-quality CPGs for RDs, it is necessary to evaluate them critically; thus, our objective was to identify CPGs for the diagnosis and management of DMD through an evaluation of methodology formulation and a comparison of clinical recommendations for a better application in clinical practice.

## 2 Materials and methods

We conducted a scoping review of CPGs for the diagnosis and management of DMD to assess their quality and compare their recommendations with a focus on developing countries. Our protocol was drafted using the Preferred Reporting Items for Systematic Reviews and Meta-analyses for scoping reviews (PRISMA-ScR) (13) and is available in the figshare database (www.prisma-statement.org/Extensions/ScopingReviews).

### 2.1 Search strategy

We performed a search to identify any potentially relevant documents from inception to February 2022 in databases (PubMed/MEDLINE, Scopus and Web of Science), guidelines formulated by organizations (National Institute for Health and Care Excellence [NICE], the Scottish Intercollegiate Guidelines Network [SIGN], Centro Nacional de Excelencia Tecnológica en Salud [CENETEC], Ministerio de Salud y Protección Social de Colombia [MINSALUD]) and repositories (Guidelines International Network (GIN) and SAGE, among others). This approach was selected as searches in databases alone may have a lower sensitivity compared to the inclusion of organizations (7). The investigation strategies were drafted by the research team and included terms related to DMD, diagnosis, and management and guideline filters. The search was conducted by three independent researchers (MC, AHR and MM).

### 2.2 Study selection

To increase consistency among reviewers, the three independent researchers (MC, AHR and MM) who performed the study selection attended a workshop on how to identify CPGs. Thereafter, the three reviewers screened the titles and abstracts and selected the full-text articles of all the publications screened; disagreements were resolved by consensus with a fourth reviewer (CAD or VVR).

All the CPGs that met the following characteristics were included: 1) CPGs based on evidence from systematic reviews, 2) the scope of which was the diagnosis and/or management of DMD, that 3) had been published no more than seven years ago. CPGs were excluded if 1) they were not published in English or Spanish or 2) the primary aim was not to inform about clinical care.

### 2.3 Data extraction

A data charting form was developed in Excel software, and two independent researchers (AHR and MM) extracted the following characteristics from the CPGs: authors, year of publication, country, target users, guideline developers, whether there was patient participation, guideline review process and the system used to grade recommendations. Additionally, recommendations related to diagnosis or management were extracted for comparison. A third reviewer (CAD) resolved disagreements in extraction by consensus.

### 2.4 Assessment of quality

To assess the quality of the CPGs selected, we used all the domains included in the Appraisal of Guidelines Research and Evaluation II (AGREE-II) (14). Each guideline was rated by five researchers (MC, ARC, MM, GAG, PMB and FCE). A grade was assigned for each item on a 7-point scale, with a score of 1 indicating that the item met none of the criteria or was very poorly reported, and a score of 7 indicated that the item met all the criteria and was well reported. We used the mean of the five raters for each item and followed the AGREE-II instrument guideline to calculate the scores for each domain (14). When a difference in two or more points for each item was found, the item was discussed to achieve consensus; disagreements were resolved by consensus with a sixth reviewer (CAD). We considered a CPG to have high quality and good quality when the total score was >60% and >80%, respectively, and we used the same cutoff for each domain of the AGREE-II instrument based on previous studies (14).

## 3 Results

### 3.1 CPG search and selection

**Figure 1:**
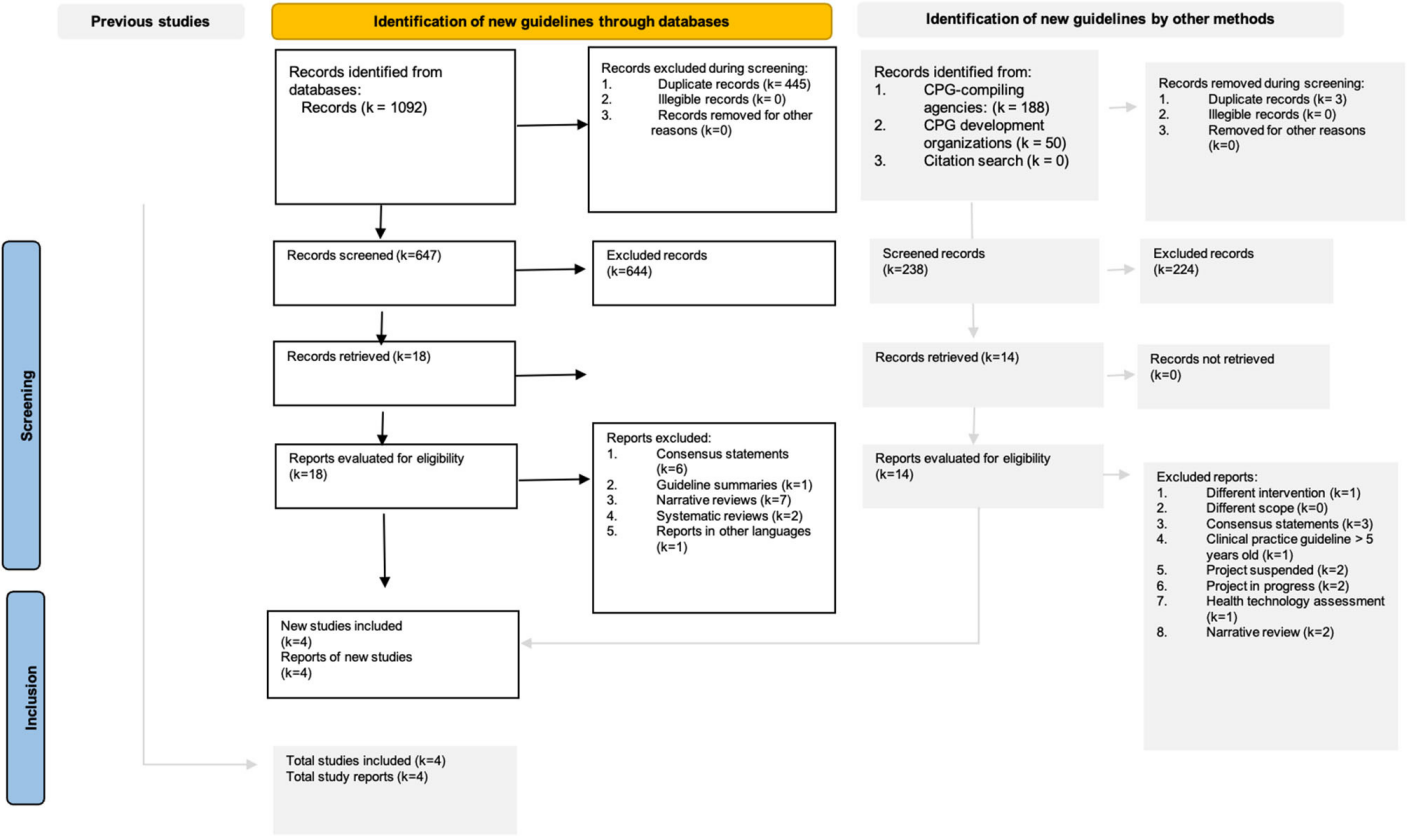
Search flowchart for the scoping review following the PRISMA guidelines.

The search strategy identified 1330 original documents (databases: 1092, CPG-compiling agencies: 188, and CPG development organizations: 50). In the title and abstract screening, 18 documents from databases met the eligibility criteria for full text review. We selected four *de novo* CPGs on the scope of diagnosis and management of DMD published or updated between 2014 and 2022 (see Figure 1). Additionally, we found nine expert consensus statements.

### 3.2 CPG characteristics

The CPGs were from England (developed by the National Institute for Health and Care Excellence [NICE]), the United States of America (developed by the American Academy of Neurology [AAN]), Colombia (developed by the Institute of Health Technology Evaluation of the Colombian Ministry of Health [IETS-MINSALUD]), and Australia (developed by the Australian National Health & Medical Research Council) (15–18). Two CPGs focused on recommendations for the diagnosis, treatment, and rehabilitation of DMD (15, 18), and one particularly focused its recommendations on nursing and other professional practices (15). The remaining two CPGs focused on treatment with ataluren (17) and corticosteroid therapy (16) (Table 1).

**Table 1:** Characteristics of the clinical practice guidelines for the diagnosis and management of Duchenne muscular dystrophy.

**Guideline developer**	**Year**	**Country**	**Included topics**	**Target users**	**Target patient or population**	**Guideline developers**	**Patient participation**	**Guideline review process**	**System used to grade recommendations**
AAN (16)	2016	USA	Treatment	Not mentioned.	People with DMD.	American Association of Neurology	No	Clinical trials	CoE
Australian CPG (15)	2020	Australia	Assessment and management	Allied health, nursing professionals, medical professionals.	Individuals with DMD of any age from the point of diagnosis.	Allied health and nursing alliance Australia and New Zealand	No	Systematic reviews and expert consensus	Delphi GRADE COSMIN
Colombian CPG (18)	2015	Colombia	Diagnosis, treatment, and rehabilitation	General clinicians, pediatricians, neurologists, physiatrists, etc.	Patients with suspected or confirmed diagnosis of DMD.	COLCIENCIA	Yes	Systematic reviews, meta-analyses, clinical trials	GRADE
NICE (17)	2016	England	Treatment	Neurologists, neuromuscular specialists, rehabilitation specialists, neurogeneticists, pediatricians and primary care physicians.	People with DMD. Parents and caretakers of people with DMD.	Evaluation committee members and NICE project team	No	Clinical trials, double blinded	Not reported

NICE, National Institute for Clinical Excellence; DMD, Duchenne Muscular Dystrophy; AAN, American Academy of Neurology; USA, United States of America; CoE, Classes of Evidence; MINSA COL, Ministerio de Salud de Colombia; GRADE, Grading of Recommendations Assessment, Development and Evaluation.

In 2/4 CPGs, the guideline developers included physicians, neurologists, nurses, rehabilitation therapists, nutritionists, and other nonhealth care professionals, such as economists (15, 18). The remaining guidelines included physicians, neurologists, nurses, and specialists in technology evaluation (16, 17). Similarly, 2/4 CPGs described in detail the participation of patients or their representatives in the development of the guidelines (15, 18).

The Grading of Recommendations Assessment, Development and Evaluation (GRADE) system was employed in two of the CPGs (Colombian and Australian CPGs) (15, 18). The Australian CPG also used the Consensus-based Standards for the selection of health status Measurement Instruments (COSMIN) and the modified Delphi process (15). The two treatment CPGs included only evidence generated from clinical trials (16, 17); one included evidence generated from randomized controlled trials (RCTs) or observational studies (15), and the remaining CPG also included evidence from systematic reviews (18).

### 3.3 Expert consensus characteristics

The majority of consensus experts were from Europe. The objectives were related to treatment, diagnosis, follow-up and patient education. Most of the studies did not report any method of reaching a consensus. However, among those that did report this, the Delphi method was the most widely used. Three articles reported a systematic search in databases (19–21). Only two studies used some method to grade the evidence (19, 20) (Table 2).

**Table 2:** Characteristics of expert consensus related to Duchenne muscular dystrophy.

**Study**	**Country**	**Objective**	**Method to achieve consensus**	**Panel participants**	**Databases used**	**Patient inclusion in consensus**	**Evidence grading system**
Aartsma-Rus (19)	The Netherlands	Diagnosis	Delphi	Medical geneticists, neurologist, neuropediatricians, patient advocates, genetic counselors	MEDLINE, Embase	No	GRADE
Araujo et al. (21)	Brazil	Diagnosis and treatment	Delphi	Neurologists, geneticists, pediatricians, cardiologists	Cochrane Library, Web of Science, Science Citation Index	No	**NR**
Bamaga et al. (22)	Saudi Arabia	Treatment during the COVID-19 pandemic	NR	Neurologists and pediatricians	NR	No	**NR**
Bernert et al. (23)	Germany	Treatment	NR	Neurologists and neuropediatricians	NR	No	**NR**
Fratter et al. (24)	UK	Diagnosis and treatment	NR	Medical geneticists	NR	No	**NR**
Jumah et al. (25)	Saudi Arabia	Diagnosis, treatment, and patient education	NR	Neurologists, cardiologists, geneticists	NR	No	**NR**
Osorio et al. (20)	Spain	Diagnosis, treatment and follow-up	NR	Neurologists, neuropediatricians, rehabilitators	MEDLINE/PubMed, Cochrane Library, Google Scholar	No	**AAN criteria**
Quinlivan et al. (26)	UK	Treatment	Unanimous agreement	Neurologists, cardiologists, endocrinologists, clinical psychologists	NR	Yes	**NR**

GRADE, Grading of Recommendations Assessment, Development and Evaluation; NR, Not reported; AAN, American Academy of Neurology.

### 3.4 Assessment of quality

**Figure 2:**
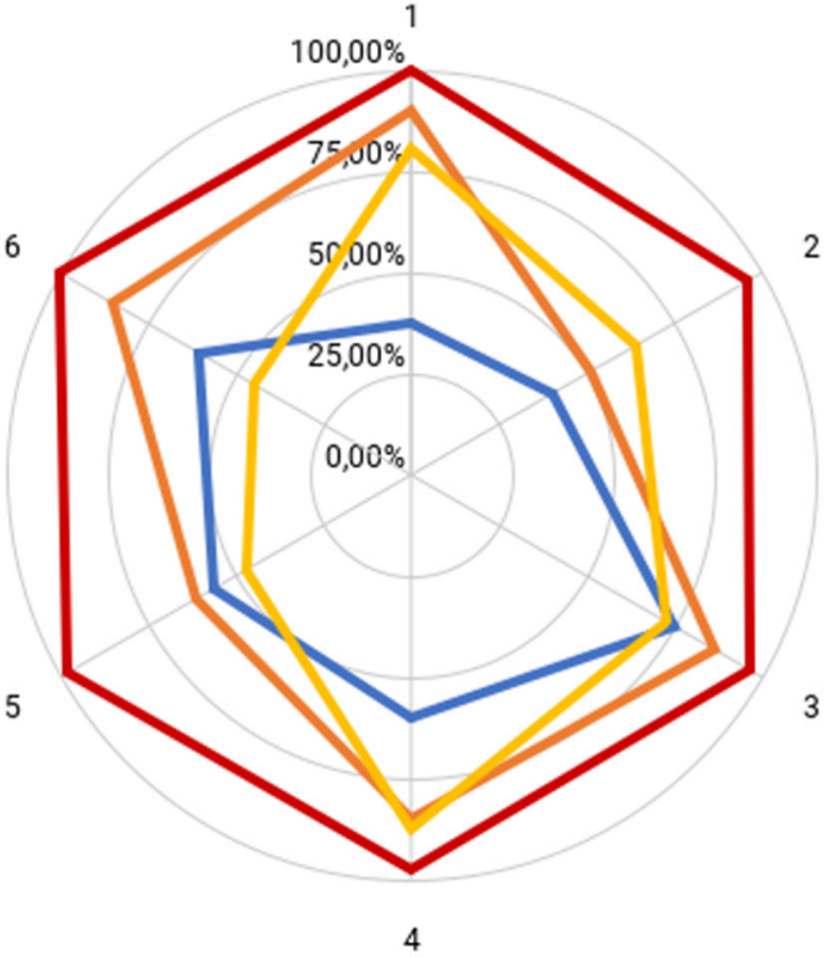
AGREE-II domain scores for included CPGs. NICE (blue), AAN (orange), Colombian CPG (red), Australian CPG (yellow). 1: Scope and purpose; 2: Stakeholder involvement; 3: Rigor of development; 4: Clarity and presentation; 5: Applicability; 6: Editorial independence.

The AGREE-II domain scores for each guideline are shown in Table 3. The Colombian CPG had a high quality and showed the highest overall assessment score (mean: 97.9%; range: 95.8%-100%) (18). Additionally, the AAN (mean: 76.6%; range: 51.4%-90.2%) (16) and Australian CPGs (mean: 66%: range: 44%-87.5%) (15) showed good quality. The NICE (mean: 54.8%; range: 37.5%-75%) (17) guideline showed lower overall scores. Figure 2 graphically compares the AGREE-II domain scores of the included CPGs.

**Table 3:** Quality appraisal of Clinical Practice Guidelines for Duchenne muscular dystrophy using the AGREE-II instrument.

**CPG**	**AGREE-II domains**						
	**1**	**2**	**3**	**4**	**5**	**6**	**Overall assessment**
NICE, 2016	37.5%	40.2%	**75%**	59.7%	56.2%	**60.4%**	54.8%
AAN, 2016	**90.2%**	51.4%	**86.5%**	**84.7%**	**61.5%**	**85.4%**	**76.6%**
Colombian CPG, 2015	**100%**	**95.8%**	**96.4%**	**97.2%**	**97.9%**	**100%**	**97.9%**
Australian CPG, 2020	**80.6%**	**63.9%**	**72.9%**	**87.5%**	46.9%	44%	66%

NICE, National Institute for Clinical Excellence; AAN, American Academy of Neurology; MINSA COL, Ministerio de Salud de Colombia; 1: Scope and purpose; 2: Stakeholder involvement; 3: Rigor of development; 4: Clarity and presentation; 5: Applicability; 6: Editorial independence.

Values >80% are bolded and represent good quality.

### 3.5 Diagnosis

Regarding CPG recommendations, the Colombian guideline had a wider scope and provided recommendations for the use of clinical criteria and the use of the measurement of serum creatinine kinase in the initial diagnostic approach for patients suspected of having DMD. Finally, the use of Western blotting, immunohistochemistry, and multiplex ligation-dependent probe amplification (MLPA) was recommended to confirm the diagnosis (18) (see Table 4).

**Table 4:** Summary of recommendations in published CPGs for the diagnosis and treatment of DMD.

**Diagnosis (MINSA COL)**	
•It is recommended to consider the age at onset, 3.3+1.56 years, to ask about symptoms of lower limb weakness and motor impairment, and to explore the presence of Gowers's sign and gastrocnemius pseudohypertrophy to guide the clinical diagnosis of DMD (Strongly in favor, very low quality of evidence) (⊕○○○). •It is recommended to use the measurement of serum creatine kinase as part of the initial diagnostic approach for patients with a clinical picture compatible with muscular dystrophy (strong support, very low quality of evidence) (⊕○○○). •It is recommended to use electromyography for the diagnosis of primary muscle fiber disease in patients with muscle weakness, high serum creatine kinase values, and no family history of muscular dystrophy (strongly in favor, moderate quality of evidence) (⊕⊕⊕○). •It is recommended to confirm the diagnosis by Western blotting for muscle dystrophin in patients with clinical suspicion of increased creatine phosphokinase (CPK) levels or electrodiagnosis of Duchenne or Becker muscular dystrophy, provided that trained personnel are available at certified institutions (strong support, low quality of evidence) (⊕⊕○○). •In the case of inability to perform Western blotting, confirmation of the diagnosis by immunohistochemical testing for dystrophin in muscle in patients with clinical suspicion, by increased creatine phosphokinase (CPK) levels or by electrodiagnosis of Duchenne or Becker muscular dystrophy is recommended, provided there are trained personnel in qualified institutions (strongly in favor, low quality of evidence) (⊕⊕○○). •In the case of inability to perform Western blotting or immunohistochemistry tests, it is recommended to use multiplex ligation-dependent probe amplification (MLPA) to detect deletions and duplications; if these are negative, gene sequencing tests can be performed (weakly in favor, low quality of evidence) (⊕⊕○○).	
**Treatment with corticosteroids**	
**MINSA COL**	**AAN**
•Treatment with steroids, 0.75 mg/kg/day of prednisone or 0.9 mg/kg/day of deflazacort, is recommended for patients with DMD to reduce mortality, prolong independent walking ability, and reduce scoliosis progression (strongly in favor, low quality of evidence) (⊕⊕○○). •It is suggested to discuss with patients and parents the continuation of steroid treatment after loss of independent walking; its use may be justified to preserve upper limb strength, reduce scoliosis progression and delay respiratory and cardiac alterations. Medical surveillance is suggested for long-term adverse effects, including periodic ophthalmologic surveillance (weakly in favor, low quality of evidence) (⊕⊕○○). •The use of steroid treatment to reduce mortality in patients with DMD without weakness; to prolong independent walking ability; to reduce the progression of scoliosis, dyspnea, and fatigue; or to improve quality of life is not recommended (strongly against, low quality of evidence) (⊕⊕○○). •The initiation of steroid therapy for patients with a diagnosis of DMD on an individualized basis is suggested, depending on functional abilities, age (no earlier than two years of age), pre-existing factors, and when motor or skill gains stop or falls increase. The initiation of steroids should be timely once motor losses occur and should be discussed with the parents and caregivers (weakly in favor, very low quality of evidence) (⊕○○○). •Intermittent steroid therapy is suggested if there are adverse events such as weight gain more than 10% that of baseline in three months, elevated blood glucose, increased blood pressure, fractures, or another intolerable event for the patient (weakly in favor, low quality of evidence) (⊕⊕○○).	•Prednisone as an intervention for patients with DMD should be used to improve strength (B) and may be used to improve timed motor function (C), should be used to improve pulmonary function (B) and may be used to reduce the need for scoliosis surgery (C), and may be used to delay the onset of cardiomyopathy by 18 years of age (C). •Deflazacort as an intervention for patients with DMD may be used to improve strength and timed motor function and delay the age at loss of ambulation by 1.4 to 2.5 years (C), may be used to improve pulmonary function (C), may be used to reduce the need for scoliosis surgery (C), may be used to delay the onset of cardiomyopathy by 18 years of age (C), and may be used to increase survival at 5 and 15 years of follow-up (C). •Deflazacort and prednisone may be equivalent in improving motor function (C). There is insufficient evidence to establish a difference in effect on cardiac function (U). Prednisone may be associated with increased weight gain in the first years of treatment compared with deflazacort (C). Deflazacort may be associated with increased risk of cataracts compared with prednisone (C). •If patients with DMD are treated with prednisone, 0.75 mg/kg/day of prednisone should be the preferred dosing regimen (B). Ten mg/kg/weekend of prednisone is equally effective over 12 months, but long-term outcomes are not yet established. A prednisone dosage of 0.75 mg/kg/day is probably associated with significant risk of weight gain, hirsutism, and cushingoid appearance (B), with an equal side effect profile seen over 12 months with the 10 mg/kg/weekend dosing. A prednisone dosage of 0.3 mg/kg/day may be used as an alternative dosing regimen with lesser efficacy and fewer AEs (level C). A prednisone dosage of 1.5 mg/kg/day is another alternative regimen; it may be equivalent to 0.75 mg/kg/day but may be associated with more AEs (C).
**Treatment with ataluren**	
**MINSA COL**	**NICE**
•Treatment with ataluren is not recommended for patients with DMD to reduce mortality, improve quality of life, prolong independent walking ability, or reduce dyspnea and fatigue (strongly against, very low quality of evidence) (⊕○○○).	•Ataluren, within its marketing authorization, is recommended for treating Duchenne muscular dystrophy resulting from a nonsense mutation in the dystrophin gene in people aged 2 years and older who can walk. •The committee concluded that, because of the uncertainty about the clinical benefits in the relevant population in clinical practice, ataluren would represent acceptable value for money to the NHS only when it was given in the context of a managed access agreement at a price that incorporated the patient access scheme and included other financial components that reduced the total costs to the NHS.
**Management strategies-Australian CPG**	
•We suggest dietary counseling (food or supplements) to increase the intake of calcium to the age-appropriate recommended dietary intake. (⊕○○○) •We suggest serial casting for selected^*^ ambulatory patients to increase ankle dorsiflexion range of motion. (⊕○○○) •We suggest the use of handheld dynamometry to assess strength. If the necessary equipment is not available, we suggest manual muscle testing to assess strength, if the evaluator is highly skilled in this assessment and testing is conducted in a standardized manner by the same evaluator. **Reliability:** **++; Measurement error:** **+++** •We suggest exercise be encouraged for boys with DMD. (⊕○○○) •We suggest nutritional supplements be used to assist strength in ambulatory boys. (⊕○○○) •We suggest the 6-minute walk test (6MWT)† be used to assess mobility. **Internal Consistency: ?; Reliability:** **++; Measurement error:** **+++; Content validity:** **+++; Hypothesis testing:** **+; Responsiveness: ?** •We suggest the North Star Ambulatory Assessment (NSAA)† be used to assess function. **Internal Consistency:** **+++; Reliability:** **++; Content validity:** **+++; Responsiveness:** **+++; Item response theory:** **+++**	
•We suggest that ankle-foot orthoses (AFOs) not be used for ambulation due to risk of harm. (⊕○○○) •We suggest that knee-ankle-foot orthoses (KAFOs) should only be implemented with careful consideration of the high resource requirements and individual variation in values and preferences. (⊕○○○) •We suggest the Performance of the Upper Limb (PUL) assessment be used†. **Internal consistency:** **++; Reliability:** **++** •We suggest the Egen Klassifikation Scale (EK scale) † be used to assess activities of daily living in non-ambulant patients. **Reliability:** **+++; Content validity:** **+++; Criterion validity:** **++** •We suggest the adoption of the 2018 DMD Care Standards for the assessment and management of respiratory function. •We suggest skinfold measures **not** be used to estimate body composition. **Criterion validity:?** •We suggest that the Schofield weight equation be used to estimate resting energy requirements. (⊕○○○) •We suggest that gastrostomy feeding, when indicated, may be effective in improving nutritional status in patients with DMD. (⊕○○○) •We suggest the adoption of the 2018 DMD Care Standards for the assessment and management of learning difficulties. •We suggest the adoption of the 2018 DMD Care Standards for the assessment and management of behavioral difficulties. •We suggest the adoption of the 2018 DMD Care Standards for the assessment and management of the transition to adult services.

### 3.6 Pharmacological treatment

The three guidelines provided recommendations for pharmacological treatment (16–18). The Colombian CPG was broadest in scope and included recommendations for treatment with steroids, and creatine (18). The Colombian CPG recommended against the use of ataluren to reduce mortality, improve quality of life, and reduce dyspnea and fatigue (18). However, the NICE guideline evaluated the application of ataluren in patients 2 years and over who can walk, and gave a conditional recommendation in the context of a managed access agreement and other financial components that reduced the total costs to the National Institute of Health (17). Additionally, the Colombian CPG and AAN guidelines agreed on the use of prednisone or deflazacort to reduce mortality, to improve timed motor function and to reduce the progression of scoliosis (16, 18) (see Table 4).

### 3.7 Nursing and other allied health professions

The Australian CPG was the only one that provided recommendations related to health assessments and management strategies by nursing and other allied health professions for patients diagnosed with DMD. The key allied health professions relevant to this CPG were dietitians, occupational therapists, physiotherapists, social workers and speech pathologists. Among its main recommendations was support and prevention by multidisciplinary teams to achieve integral attention for DMD patients (15) (see Table 4).

## 4 Discussion

### 4.1 Main findings

We found a total of four CPGs, of which only one included recommendations for diagnosis and treatment; two CPGs, a practice guideline and a health technology assessment, were focused only on corticosteroid or ataluren therapy, respectively; and one CPG included recommendations about allied health and nursing practice for the assessment and management of DMD patients (15–18).

### 4.2 Assessment of quality

In relation to the quality of guideline development, the best performing CPG was the Colombian CPG, followed by the AAN and Australian CPGs (15, 16, 18). The NICE guideline showed low overall scores in the AGREE-II domains (17). The NICE guideline obtained a low score, probably due to its narrow objectives since it was mainly an evaluation of new technology (17). When analyzing the third domain (rigor of development), 2/4 CPGs achieved a very good score (> 80%) (16, 18). The other guides obtained good scores (>60%) (15, 17). Our results indicate that, in general, the CPGs had adequate methodological rigor, which could mean that they were based on the best available evidence identified from the SR process in addition to incorporating other multiple criteria, which was adequate to make trustworthy recommendations (27–29).

Another important aspect to consider is the system used to make recommendations. In this regard, 2/4 CPGs clearly described the methodology used step by step (Colombian and Australian guidelines) (15, 18). The AAN guideline reported the system used to grade recommendations but did not clearly describe the entire process. The application of a systematic review process guarantees the use of the best evidence but not the development of trustworthy recommendations from this evidence. GRADE is the most accepted approach for this, as it assesses the body of evidence using different criteria such as study design, risk of bias, imprecision, indirectness, inconsistency, and others to increase or reduce the certainty of evidence (30). This allows for a simple and transparent grading of recommendations that can effectively contribute to improving clinical practice, even in the scenario of limited evidence for RDs (31, 32).

### 4.3 Stakeholder involvement

On the one hand, stakeholders are defined as “individuals, organizations, or communities that have a direct interest in the process and outcomes of a project, research, or policy” (33). In CPGs, the patients are of particular importance, as they are affected by the diseases, and the outcomes investigated are a key component in the applicability of the guidelines. Patients can help to identify research questions and important issues in their health care and include perspectives from different settings (16, 34, 35). Nevertheless, we found that 2/4 CPGs reported in detail the participation of the patients in the review of the scope, identification and grading of outcomes and in the review of the draft (Colombian and Australian CPGs) (15, 18). Both guidelines achieved a score >60% in this domain.

Regarding the other guidelines, the NICE guideline includes a patient advocate as a specialized technologies evaluation committee member (36). Additionally, the process manual to develop clinical practice guidelines of the AAN recommends including at least one patient in formulating the clinical question and review of the draft (37). However, we did not find details of the participation of patients and/or their representatives in the DMD guidelines developed by these institutions (16, 17). The NICE and AAN guidelines probably did not obtain adequate quality scores in the stakeholder involvement domain due to their limited scope or because a professional association developed them.

### 4.4 Clarity, presentation, and editorial independence

Most of the guidelines reported easily identifiable, specific, and unambiguous recommendations. This helps in the uptake of guidelines by target professionals and patients (38). In addition, the Colombian CPG presented a flowchart for patients with suspected DMD and for treatment with steroids and deflazacort (18).

The editorial independence domain is important because funding and conflicts of interest (CoIs) can introduce bias in developing recommendations (39). We found that the CPGs were supported by transparent institutions that reported financing. However, not all the guidelines described how potential CoIs influenced and how they were controlled for in the elaboration of the recommendations, which is a common limitation (40, 41).

### 4.5 Diagnosis and treatment recommendations

Only the Colombian CPG included diagnosis in its scope and therefore included recommendations regarding the types of diagnostic tests available for DMD. In addition, it mentioned an economic evaluation within the CPG, recommending the use of Western blotting, followed by immunohistochemistry and finally MLPA. However, given that different techniques may be available in each country, factors such as training and standardization should be involved in deciding the techniques required for diagnosing DMD (31).

Two CPGs recommended corticosteroids as the standard of care for DMD due to their significant benefits (16, 18). Nevertheless, glucocorticoids (prednisone and deflazacort) are associated with notorious adverse effects (AEs), such as weight gain, cushingoid appearance, growth retardation/failure to thrive, behavioral changes, fractures due to osteoporosis, cataracts, and skin fragility (40, 42). For this reason, intermittent therapy has been investigated as an alternative; unfortunately, one randomized controlled trial did not show significant improvement the comparison between daily and intermittently 10 days on and 10 days off (43). Despite this, due to an outdated state of the available evidence, the Colombian CPG recommends intermittent therapy when the patient develops AEs (17, 18), whereas the AAN CPG considers that the evidence is insufficient (16). Additionally, new alternatives are currently under investigation, such as vamorolone, a steroid analog, where doses of 2 and 6 mg/kg per day showed improvements in multiple functional end points over the 24-week treatment period (44).

Ataluren, a small orally bioavailable molecule that induces ribosomal read-through of nonsense mutations, was conditionally approved by the European Medicines Agency in 2015 (45), but in 2023 recently recommended non-renewal of authorization Translarna (ataluren) (46); besides, has not yet been approved by the Food and Drug Administration (47). Since the Colombian CPG was developed in 2014, it did not include the two new RCTs on ataluren (48, 49). This could explain why this CPG recommended against treatment with ataluren (18). Although Ataluren treatment is controversial, ongoing studies are recruiting patients for long-term efficacy and safety. In addition, the drug has demonstrated better benefits than placebo, and with standard of care is even better, albeit with limited evidence (50, 51). For example, the NICE guideline concluded that ataluren represents an acceptable option only in the context of a managed access agreement (MAA) based on clinical results (17, 52). However, by certain organizations exist restriction for the use of the innovative technology when it is expensive and exist uncertainty about it effectiveness and security. Consequently, patients with DMD are left without appropriate treatment options (53–55).

### 4.6 Nursing and other allied health professions

DMD is a disease characterized by progressive muscle degeneration and functional losses. Thus, it is important to develop supportive and prevention recommendations by multidisciplinary teams to achieve integral attention for DMD patients (56). However, we found only one CPG, the Australian CPG (15), that focused on health issues not covered by guidelines aimed at diagnosis and/or medical treatment. This guideline had a broad scope, including dietary counseling, rehabilitation, respiratory function, behavioral or learning difficulties management, etc. In addition, the users of the guideline were professionals from different areas of health. This type of scope is novel but very important for the proper care of DMD patients. Further studies could include its conceptual framework within the development methodology.

### 4.7 Expert consensus

Most experts agreed with CPGs about the recommendations for diagnosis and treatment. Apart from these, some suggested that family education can help address the problem of stigma at home and in society (25). Additionally, they indicated regular nutritional monitoring and diet adjustments (20).

### 4.8 Formulating recommendations for rare diseases

The paucity of evidence is a serious concern for RDs. The CPGs we identified based their recommendations on systematic reviews of clinical trials and cost-effectiveness studies. They did not use other types of evidence, such as observational studies for treatment, real-world data, or qualitative or narrative studies, which are important in the field of RDs (5, 31). Furthermore, RD studies, when available, may have multiple limitations (31). Some of these limitations include the rarity of RCTs, study heterogeneity, and challenges in patient enrollment, among others (31, 54).

Researchers have developed methodologies for eliciting and synthetizing evidence for CPGs on RDs. The RARE-Best Practices Working Group shares methodological knowledge for guideline development in the field of RDs (31, 32). First, intentional and systematic gathering of observational evidence from experts is a more efficient and transparent alternative to informal “around the table discussion” by guideline panels (5). Additionally, indirect evidence from other diseases and access to a patient registry of the target disease to complement published evidence with low certainty is a feasible alternative (5). Finally, in this context, qualitative research could be a feasible source of information (5). However, these registries were not implemented in the CPGs identified, as they were published previously or did not incorporate the GRADE methodology. Future guidelines should consider these recommendations during development because they can increase reliability.

### 4.9 Limitations

One of our study limitations was that only guidelines published in English or Spanish were collected, and thus, our findings may not be representative of CPGs published in other languages. Although most organizations publish their guidelines in English, one of the CPGs was excluded because it was only available in Chinese. Further studies should consider evaluating the quality of CPGs published in other languages. Although the CPGs originated from high-income countries, exist different population in patients with DMD around the world with disparities in healthcare access, medication and accurate diagnosis availability. It is imperative to consider this heterogeneity in DMD populations when its developing EBG to incorporate implementation considerations (57).

### 4.10 Conclusions

We performed a scoping review of CPGs for DMD, identifying four guidelines: one for the diagnosis and management of DMD, two for ataluren and corticosteroid therapy, and one focused on allied health professionals and nursing. In addition, nine expert consensuses were identified. All the guidelines showed high or good performance in the rigor of development. Recommendations were coherent across CPGs on steroid treatment, except for minor differences in dosing regimens. However, there was disagreement regarding the use of ataluren, mainly due to the absence of evidence at the time of publication. Finally, no diagnostic or treatment guidelines have been published or actualized in the last 5 years, and we recommend updating the existing guidelines and implementing a reliable methodology in their development.

## Author contributions

MM: Conceptualization, Formal analysis, Methodology, Writing—original draft, Writing—review & editing. AR-C: Conceptualization, Formal analysis, Methodology, Writing—original draft, Writing—review & editing. FC-E: Conceptualization, Formal analysis, Investigation, Methodology, Writing—original draft, Writing—review & editing. AH-R: Investigation, Methodology, Writing—original draft. GA-G: Investigation, Methodology, Validation, Writing—original draft. MC-L: Investigation, Methodology, Writing—original draft. PM-B: Data curation, Validation, Writing—original draft. VV-R: Formal analysis, Investigation, Methodology, Software, Validation, Writing—original draft, Writing—review & editing. PM-E: Supervision, Validation, Visualization, Writing—original draft, Writing—review & editing. CA-D: Conceptualization, Funding acquisition, Investigation, Project administration, Resources, Validation, Visualization, Writing—original draft, Writing—review & editing.
